# Magnetisation switching dynamics induced by combination of spin transfer torque and spin orbit torque

**DOI:** 10.1038/s41598-022-07277-2

**Published:** 2022-03-01

**Authors:** Andrea Meo, Jessada Chureemart, Roy W. Chantrell, Phanwadee Chureemart

**Affiliations:** 1 Magnetic information storage technology (MINT) research unit, Department of Physics, Mahasarakham University, Mahasarakham, 44150 Thailand; 2 Department of Physics, University of York, York, YO10 5DD UK

**Keywords:** Mathematics and computing, Nanoscience and technology, Physics

## Abstract

We present a theoretical investigation of the magnetisation reversal process in CoFeB-based magnetic tunnel junctions (MTJs). We perform atomistic spin simulations of magnetisation dynamics induced by combination of spin orbit torque (SOT) and spin transfer torque (STT). Within the model the effect of SOT is introduced as a Slonczewski formalism, whereas the effect of STT is included via a spin accumulation model. We investigate a system of CoFeB/MgO/CoFeB coupled with a heavy metal layer where the charge current is injected into the plane of the heavy metal meanwhile the other charge current flows perpendicular into the MTJ structure. Our results reveal that SOT can assist the precessional switching induced by spin polarised current within a certain range of injected current densities yielding an efficient and fast reversal on the sub-nanosecond timescale. The combination of STT and SOT gives a promising pathway to improve high performance CoFeB-based devices with high speed and low power consumption.

## Introduction

In today’s information based society enormous amounts of data are stored and processed daily. Current spintronic devices such as spin transfer torque magnetic random access memories (STT-MRAMs) are among the most promising emerging memory technologies. STT-MRAMs have been considered as candidates for universal memories thanks to their potential for fast read/write operations, low power consumption, non-volatility and durability^1–6^. STT-MRAMs are based on magnetic tunnel junctions (MTJs), that is a trilayer structure consisting of two conductive ferromagnets and a thin insulator in the middle, usually CoFeB-based and MgO respectively^7^. One ferromagnet, termed the reference layer (RL), has pinned magnetisation and polarises the spin current flowing into the layer. The other ferromagnet, whose magnetisation can be freely rotated, is known as the free layer (FL). It can be reversed by a large enough excitation of the spin polarised current. In the case of STT-MRAM this is achieved by injecting a perpendicular current into the MTJ and exploiting the torque arising from the s-d exchange interaction between the conduction electrons and the local magnetisation. The information is retrieved by sensing the magnetisation state of the latter via the tunnel magnetoresistance (TMR) effect. These devices can yield fast switching, hence fast writing operations, on the order of a few nanoseconds. However, to achieve faster switching large current densities are required^2,3,5,8^. This undermines the potential for low power applications. Moreover, since the injected current and the magnetisation of the free layer share the same direction at rest, it is necessary to induce misalignment of the magnetisation in order to have a non-zero torque. This might cause long incubation times thereby reducing the speed. STT-MRAM is a two terminal device, i.e. the same electrical path is used to write and read. This offers the opportunity of reducing the cell dimension of the memory, thus going beyond the current silicon-based technology. On the other hand, the thin oxide that serves as tunnelling barrier in the MTJ may suffer damage when large current densities or voltages are applied. This can cause the breakdown of the device and result in durability issues^5,8^.

Apart from the conventional STT switching by injecting a current perpendicular to the MTJ, the current-induced magnetisation switching can be achieved by spin-orbit torque (SOT), in SOT-MRAM devices^8–10^. SOT can be generated by various mechanisms^11^ such as spin Hall effect (SHE), Rashba effect, inverse spin Galvanic effect, anomalous Hall effect and interfacial spin-precessional scattering. SOT has recently sparked interest in both theoretical and experimental research due to its application diversity. For example, SOT switching driven by mechanism of spin-processional scattering and anomalous Hall effect have been experimentally demonstrated by Baek et al.^12^ and Ma et al.^13^ respectively. Interestingly, generation of unconventional spin current in symmetry-reduced system such as CuPt^14^ and spin-orbit torque generated by the broken magnetic symmetry in the antiferromagnetic IrMn$_3$^15^ have been reported. One of the most known and studied mechanisms is SHE, which arises when a current is injected into a non-magnetic metal and spin up and spin down electrons are scattered to opposite directions. This creates an accumulation of spin density in the directions orthogonal to the current flow. This effect is caused by spin-orbit coupling and thus is larger in heavy metals^11^. SOT-MRAMs are devices based on MTJs that utilise SOT for the writing process, while using the TMR effect to retrieve the information as in STT-MRAM. SOT can be achieved by placing a non-magnetic heavy metal (HM), Pt, Ta or W generally, in contact with the ferromagnetic layer and injecting an in-plane electrical current into the HM. Spin-orbit coupling is responsible for the generation of a pure spin current orthogonal to the current direction. This is absorbed in the ferromagnet and exerts a torque on the magnetisation of the ferromagnet that can reverse its polarisation^11^. Due to the nature of the process, reversal occurs in a few hundreds of picoseconds. SOT does not suffer the incubation time issue, however it requires a way to break the symmetry so that the magnetisation ends in the desired final state. The traditional approach is to apply an in-plane magnetic field in the direction of the injected current density. The application of a field has the downside of increasing the power consumption of the device and it might affect the magnetisation of the MTJ itself or nearby cells. Nonetheless, SOT-MRAMs are characterised by a slightly lower power consumption than STT-MRAM^16,17^. SOT-MRAM is a three terminal device in which the writing and reading paths are separated: the writing current is injected into the metallic contact and does not flow across the MTJ, whereas a weak current injected into the MTJ is used to read. This ensures that only weak currents cross the MTJ avoiding the risk of damaging the barrier, hence improving the endurance. Moreover, it increases the reliability by avoiding undesired writing while reading since the read and write path are separated^3,5,11,16^. This comes at the cost of larger memory cells than STT-MRAM. Nonetheless, their fast operation, on the sub-nanosecond timescale, make these devices suitable for CPU cache memories^11,16,17^.

Various alterations to the standard STT-MTJ stack^5,6^, such as dual MgO MTJs^18,19^, have been proposed. However, the major limitation affecting these solutions is the minimum MTJ size achievable. Voltage control of magnetic anisotropy (VCMA) MTJs^20,21^ offer the potential of low power magnetisation operations. In practice however, VCMA requires the assist from a charged current or a very precise control of the voltage pulses to yield a reliable deterministic switching^22^. Shape anisotropy MTJs^23–25^, whose free layer is patterned into a tall cylindrical ferromagnet, have been identified as pathway to scale the MTJ size down to or below 10 nm. However, efficient magnetisation dynamics and transport properties in such structures are yet to be fully demonstrated. To overcome the limitation of an external magnetic field in SOT-MRAMs, field-free switching has been proposed by controlling the geometry of the device^26^, introducing a tilted magnetic anisotropy^27^, using in-plane MTJs^28^ and T-type structures^29^, exploiting the exchange bias via coupling of the ferromagnetic layer with an antiferromagnet^30^, combining VCMA with SHE-induced SOT in in-plane MTJs^31^, using a magnetic hard mask to shape the SOT track^32^. However, downsides of these solutions are the scaling of the cell size and the limited efficiency^26,30^ and in some cases difficulties of integration in the current CMOS technology. Recently, Wu et al.^33^ proposed a voltage-gate-assisted SOT-MRAM that combining VCMA and SOT offers low energy consumption, fast operations and a high storage density. However, the design requires MTJ in-plane dimensions to be reduced and the writing still requires the application of an-plane magnetic field.

Another possible solution to overcome the intrinsic limitations affecting STT- and SOT-MRAMs is to combine these two effects in a single device^11,34–43^. This opens two scenarios: STT assisted by SOT and SOT assisted by STT. In the first case SOT acts by inducing an initial misalignment of the magnetisation of the ferromagnet. This would eliminate the incubation time seen in STT-MRAMs leading to significant reduction of the switching time. When STT assists SOT-driven dynamics, STT can provide the necessary bias to break the symmetry and provide deterministic switching of the magnetisation. This removes the necessity of an external field or to resort to engineered magnetic structures^8,11,28,44–46^ reducing the required current density. Most of the studies on SOT-MRAM assisted by STT focused on three-terminal devices^11,37,45,46^, even though there are reports of two-terminal devices^38,39^. SOT-assisted STT-MRAMs are investigated in Ref.^34–36,38,41^ showing improvements in the switching time and power consumption, however in these works long switching times are considered. Wang and collaborators^40^ propose a toggle spin torque MTJ where SOT and STT current pulses are sequentially applied, whilst Xu et al.^42^ propose a triple level cell combining STT and SOT-based MTJs where the writing mechanism is achieved in two steps.

To develop the performance of MTJ, it is important to understand the magnetisation dynamics of the magnetic material used in devices, since the critical current density for the reversal process, operating speed and power consumption are closely related to the dynamic behaviour. This work aims to develop a computational model which combines the atomistic spin model and a spin transport model for MTJ design. The majority of MTJ models employed to study magnetic systems are generally based on the macrospin and micromagnetic formalism^3,34,35,38,41,47–53^. These face limitations when dealing with the miniaturisation and down-scaling of the the device dimensions to few nanometre due to the continuum assumption that the magnetisation varies smoothly with position. In particular the macrospin approach assumes uniform magnetic properties over the whole ferromagnetic layer and therefore it is constrained to uniform magnetic configurations. On the other hand, the discrete nature of the atomistic model allows to have spatially varying magnetic properties over different lattice sites, something hard for micromagnetic simulations even with the finest discretisation, enabling the description of interfaces, surface and finite size effects. These features make possible to describe the anisotropy in CoFeB/MgO MTJs more accurately, one of the most important parameter for technological applications, that arises from and is localised at the CoFeB|MgO interface or the increase of the magnetic damping at the same interface^54^. For these reasons, in this work we investigate the influence of the combined STT and SOT phenomena on the magnetisation switching dynamics of CoFeB/MgO-based MTJs at the atomistic level, whose details can be found in the “Methods” section. The results offer a new pathway to control the magnetisation in MTJs by injecting both an in-plane current into the HM adjacent to the ferromagnetic layer and a current perpendicular to the MTJ. This is shown to give rise to higher efficiency of spin torque switching.

**Table 1:** List of parameters used in the simulations.

	CoFeB-bulk	CoFeB-int	Unit	Parameter name
$J_{ij}$	${7.735\times 10^{-21}}$	${1.547\times 10^{-20}}$	$\text {J}\, \text {link}^{-1}$	Exchange energy constant
$k_{\mathrm {u}}$	0	${1.35\times 10^{22}}$	$\text {J}\,\text {atom}^{-1}$	Uniaxial anisotropy energy constant
$\mu _{\varvec{s}}$	1.6	1.6	$\mu _{B}$	Atomic spin moment
$\lambda$	0.003	0.11		Gilbert damping
$\vartheta _{\mathrm {SH}}$	0.11	0.11		Spin Hall angle
$\beta$	0.56	0.56		Conductivity spin polarisation
$\beta ^{'}$	0.72	0.72		Diffusion spin polarisation
$\lambda _{\mathrm {sdl}}$	12	12	nm	Spin diffusion length
$D_0$	0.001	0.001	$\text {m}^{2}\text {s}^{-1}$	Diffusion constant
$J_{\mathrm {sd}}$	$1.6\times 10^{20}$	$1.6\times 10^{-20}$	J	*s*-*d* exchange energy constant
$m_{\infty }$	$2.62\times 10^{8}$	$2.62\times 10^{8}$	$\text {Cm}^{-3}$	Equilibrium spin accumulation

## Results

**Figure 1:**
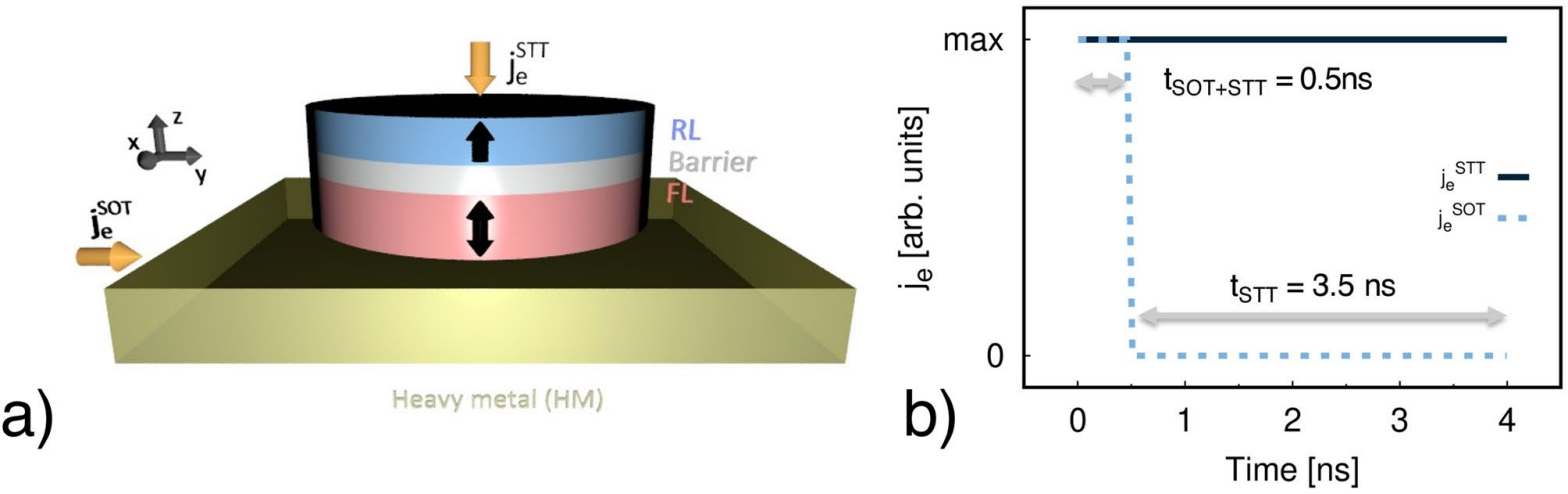
(**a**) Sketch illustrating the investigated system. Brass colour depicts the heavy metal (HM), red and light blue represent the free layer (FL) and reference layer (RL) of the MTJ respectively, light grey refers to the MTJ barrier. $j_e^{\mathrm {SOT}}$ and $j_e^{\mathrm {STT}}$ (yellow arrows) are the current densities injected in the HM and MTJ, respectively. $j_e^{\mathrm {STT}}$ is injected along the *y*-direction, $j_e^{\mathrm {STT}}$ is perpendicular to the structure along the *z*-direction. (**b**) Simulation protocol: initially both $j_e^{\mathrm {SOT}}$ (dashed light blue line) and $j_e^{\mathrm {STT}}$ (solid black line) are injected for 0.5 ns. After 0.5 ns $j_e^{\mathrm {SOT}}$ is switched off and only $j_e^{\mathrm {STT}}$ is injected into the system in the next 3.5 ns.

In this work we simulate the magnetisation dynamics induced by a combination of STT and SOT in CoFeB/MgO/CoFeB MTJs by means of an atomistic spin model. Figure 1a presents a sketch of the investigated system. We parametrise CoFeB (1.0 nm, RL)/MgO (0.85 nm, Barrier)/CoFeB (1.3 nm, FL) as done in Ref.^55^, where the number in brackets is the thickness, and FL and RL refer to free and reference layer, respectively. A list of the parameters used in the simulations is presented in Table 1 and details of the model and parameters are given in the “Methods” section. To simulate the magnetisation dynamics induced by the combination of SOT and STT, we consider a MTJ of diameter ranging from 5 to 30 nm and perform simulations at a temperature of 0 K. However, the discussion presented here focuses on the properties of MTJs with diameter of 20 nm, since this is the dimension most likely to be used in real applications. The protocol, sketched in Fig. 1b, is such that in the initial 0.5 ns both $j_e^{\mathrm {SOT}}$ and $j_e^{\mathrm {STT}}$ are injected. After 0.5 ns, $j_e^{\mathrm {SOT}}$ is switched off while $j_e^{\mathrm {STT}}$ continues to be injected into the system for the next 3.5 ns. As shown in Fig. 1a, the current density $j_e^{\mathrm {SOT}}$ is injected into the HM along the positive *y*-direction. As a consequence the spin polarisation $\varvec{\hat{\sigma }}$ is directed along $-x$. The transverse current density $j_e^{\mathrm {STT}}$ used to induce STT is injected perpendicularly into the RL along the *z*-axis. The system is initialised in an antiparallel state with the RL magnetisation aligned along the positive *z*-axis and the FL polarised along the opposite direction. We would like to point out that the realisation of SOT and STT-induced magnetisation reversal in MTJs with current perpendicular to the stack where, in absence of the HM, SOT is solely provided by Rashba effect, is not a viable solution. In fact, to have a non-zero Rashba effect the current should be applied in an in-plane configuration, as it has been reported in literature^56,57^, due to symmetry requirements. Thus this solution cannot be applied to the MTJ stack considered in the present study. To evaluate the dynamical response of the combined SOT+STT protocol we perform three investigations triggered by: (A) pure spin transfer torque, (B) pure spin orbit torque and (C) the combination of both STT and SOT as detailed in the following sections.

### Pure spin transfer torque

**Figure 2:**
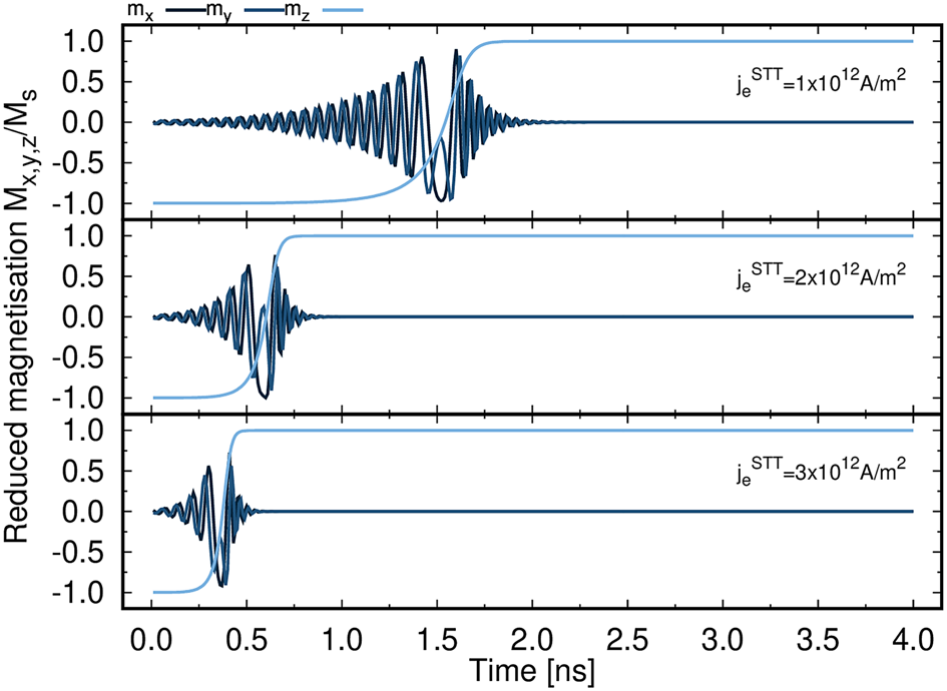
Time evolution of the magnetisation components of a 20 nm diameter MTJ for $j_e^{\mathrm {STT}}$ ${1\times 10^{12}, \, 2\times 10^{12} \, \text {and} \, 3\times 10^{12}} \, \text {Am}^{-2}$.

We initially investigate the effect of the transverse injected current density $j_e^{\mathrm {STT}}$ in the absence of the in-plane current $j_e^{\mathrm {SOT}}$ injected into HM. To ensure a minimum torque acting on the system the FL magnetisation is tilted ${1}^{\circ }$ from the *z*-axis. Charge currents with different densities are perpendicularly injected into the MTJ structure. As demonstrated in Fig. 2, we observe the precessional motion of magnetisation in the *xy* plane before reaching the steady state in the *z* direction. Increasing charge current density results in higher spin transfer torque acting on the magnetisation and subsequently reduces the switching time. In the case of pure STT the system exhibits the expected dynamics with a slow rise of the precession until $M_z$ changes sign, followed by a more strongly damped motion due to the STT symmetry. As discussed in Ref.^58^, STT-induced magnetisation dynamics is characterised by a rotation of the in-plane component and by either coherent reversal or non-collinear reversal of the out-of-plane component. The former is always observed and is caused by the STT symmetry, the latter depends on the size and magnitude of $j_e^{\mathrm {STT}}$. The excitation modes, introduced in the Method section, show an overall dominant coherent character of the dynamics in these cases, with excitation of the vortex and antivortex modes^58^ for strong $j_e^{\mathrm {STT}}$ and large diameters. It is worth mentioning that when STT is modelled via the spin accumulation, the reversal mechanism is coherent up to diameters of 20 nm. Only for large $j_e^{\mathrm {STT}}$ non-collinear modes are weakly excited, with the usual transition from a vortex to an antivortex mode. This differs slightly from the case of a macrospin-derived approach, as in Ref.^58^, where for large $j_e^{\mathrm {STT}}$ the reversal can exhibit strong excitations of the non-collinear modes at such dimensions. This can likely be ascribed to the missing in-plane spacial dependence and angular dependence in the latter approach, which can lead to an overestimation of the torque acting on the system.

**Figure 3:**
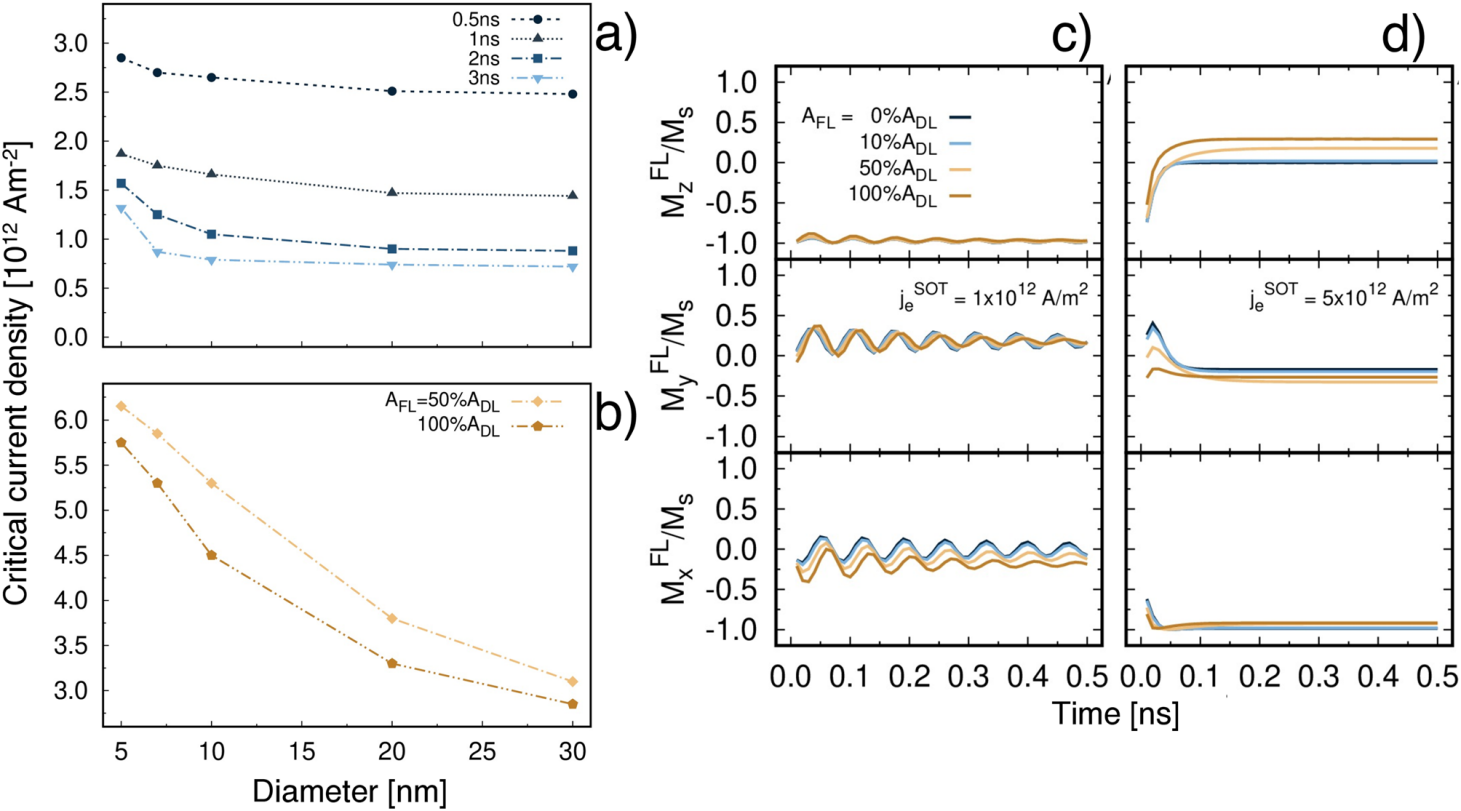
(**a**) Plot of the critical current density for STT switching $j_c^{\mathrm {STT}}$ as a function of MTJ diameter for different pulse widths. (**b**) Plot of the critical current density for SOT switching $j_c^{\mathrm {SOT}}$ as a function of different strengths of the SOT field-like component ($A_{\mathrm {F}}$) with respect to the damping-like term ($A_{\mathrm {D}}$). For $A_{\mathrm {F}} ={0}{\%}$ and 10% $j_c^{\mathrm {SOT}}$ could not be determined. Time evolution of the magnetisation components of a 20 nm diameter MTJ as a function of $A_{\mathrm {F}}$ for $j_e^{\mathrm {SOT}}$ ${1\times 10^{12}} \, \text {Am}^{-2}$ (**c**) and ${5\times 10^{12}} \, \text {Am}^{-2}$ (**d**) in absence of $j_e^{\mathrm {STT}}$.

We can extract the critical current density for STT switching $j_c^{\mathrm {STT}}$ as the minimum $j_e^{\mathrm {STT}}$ that yields the reversal of the magnetisation within a fixed time. Since it is expected that $j_c^{\mathrm {STT}}$ varies with the pulse length, we extract $j_c^{\mathrm {STT}}$ for 0.5, 1.0, 2.0 and 3.0 ns and we plot it as a function of MTJ diameter in Fig. 3a. To obtain reversal in less than a nanosecond large current densities need to be injected into the MTJ, on the order of ${2.5\times 10^{12}} \; \text {Am}^{-2}$. Such a requirement can be halved or more if the pulse is applied for 2 ns. Small diameters are characterised by longer incubation time, as observed in Ref.^58^, resulting in larger critical current densities. Thus, STT-induced precessional switching sets a limitation to size scaling in a low temperature regime.

### Pure spin orbit torque

We next investigate the effect of the in-plane current density $j_e^{\mathrm {SOT}}$ in the absence of transverse $j_e^{\mathrm {STT}}$ to evaluate the contribution of SOT to the magnetisation dynamics. We vary $j_e^{\mathrm {SOT}}$ and we study the switching dynamics for different strengths of the field-like component of SOT ($A_{\mathrm {F}}$) with respect to the damping-like term ($A_{\mathrm {D}}$). The magnetisation response induced by SOT is shown in Fig. 3c,d for a low and high $j_e^{\mathrm {SOT}}$, respectively. For $j_e^{\mathrm {SOT}}$ less than ${1\times 10^{12}} \; \text {Am}^{-2}$ there is no precession of the magnetisation in the FL. For increasing magnitudes of $j_e^{\mathrm {SOT}}$ weak precession of the magnetisation can be observed, however no reversal occurs, as shown in Fig 3c for $j_e^{\mathrm {SOT}} ={1\times 10^{12}} \; \text {Am}^{-2}$. As $j_e^{\mathrm {SOT}}$ increases further, the magnetisation tends to align along the direction of $\varvec{\hat{\sigma }}$, $-x$ in our case, within less than 0.1 ns. Afterwards the magnetisation is in equilibrium and only slow changes in the magnetisation occur. In this case we do not observe the precession of the magnetisation as the system reaches equilibrium before one precessional cycle is completed. A comparison of the different curves in Fig. 3c,d shows that a non-zero $A_{\mathrm {F}}$ hinders the precession of the magnetisation and for large $j_e^{\mathrm {SOT}}$ results in a tilt of the magnetisation towards the *z*-axis. However, this misalignment from the in-plane direction alone cannot yield the complete reversal of the magnetisation. Pure SOT dynamics is characterised by coherent rotation of the magnetisation, even for the largest size investigated. We attribute the coherent behaviour to the symmetry of SOT that tends to favour in-plane configurations and to suppress higher excitation modes.

In the case of pure SOT, since we cannot use the switching time to define the critical current density $j_c^{\mathrm {SOT}}$, we consider the minimal $j_e^{\mathrm {SOT}}$ that yields a reduced magnetisation $m_z=M_z/M_{\mathrm {s}}$ larger than 0.7, as proposed in Ref.^41^. The plot of the size dependence of $j_c^{\mathrm {SOT}}$ as a function of different $A_{\mathrm {F}}$ strengths is presented in Fig. 3b. $j_c^{\mathrm {SOT}}$ decreases for increasing diameters, in a similar trend to $j_c^{\mathrm {STT}}$, due to the magnetostatic contribution. However, we cannot extract $j_c^{\mathrm {SOT}}$ for $A_{\mathrm {F}} ={0}{\%}$ and ${10}{\%}A_{\mathrm {D}}$ since the magnetisation lies in-plane at the end of the pulse, as clear from Fig. 3d.

### Effect of combined spin transfer torque and spin orbit torque

**Figure 4:**
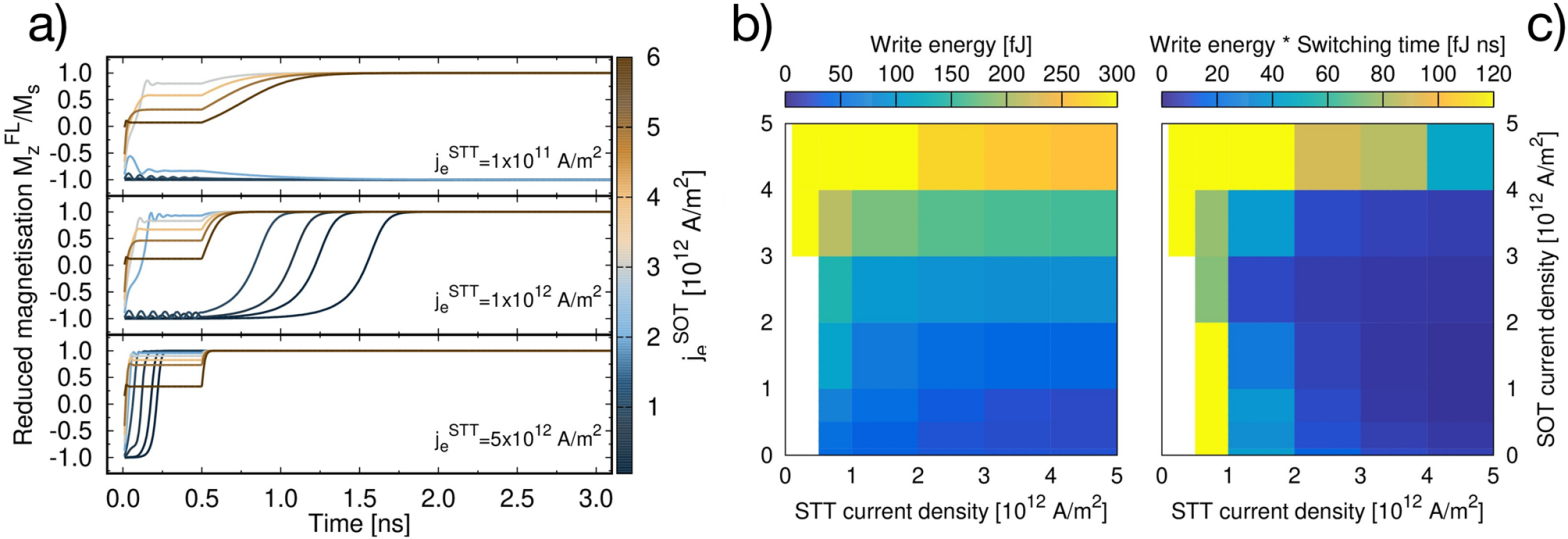
(**a**) Time evolution of the reduced *z*-component of the magnetisation as a function of $j_e^{\mathrm {SOT}}$ for $j_e^{\mathrm {STT}} =1\times 10^{11}, 1\times 10^{12}\text { and }5\times 10^{12} \; \text {Am}^{-2}$. In this case $A_{\mathrm {F}} =A_{\mathrm {D}}$ and the diameter is 20 nm. (**b**) Write energy and c) write energy $\times$ total switching time as a function of $j_e^{\mathrm {STT}}$ and $j_e^{\mathrm {SOT}}$ for $A_{\mathrm {F}} =A_{\mathrm {D}}$ and diameter of 20 nm. The palette represents the energy in (**b**) and energy $\times$ switching time in (**c**), and ranges from blue (low) to yellow (high). White regions correspond to combinations of $j_e^{\mathrm {STT}}$ and $j_e^{\mathrm {SOT}}$ that yield no switching.

Having fully characterised the dynamical response to STT and SOT individually, we now study the combination of both the effects using the protocol shown in Fig 1b. Our results show that STT can yield reversal, but this occurs in a few nanoseconds. On the other hand, a rapid magnetisation response of a few hundreds of picoseconds can be achieved through the SOT, with the caveat that an additional contribution is required to obtain a full and deterministic reversal of the magnetisation. Hence, using the SOT rapidly to develop a transverse magnetisation to assist the STT might be expected to yield a more efficient switching dynamics than for the two cases separately. Figure 4a shows the time evolution of the reduced *z*-component of the magnetisation as a function of $j_e^{\mathrm {SOT}}$ for $j_e^{\mathrm {STT}}$ = ${1\times 10^{11}, 1\times 10^{12}\text { and }5\times 10^{12} \; \text {Am}^{-2}}$ with $A_{\mathrm {F}} =A_{\mathrm {D}}$. Similarly to the results in presented in Ref.^41^, we can distinguish different scenarios depending on the combination of $j_e^{\mathrm {STT}}$ and $j_e^{\mathrm {SOT}}$:

1. Small $j_e^{\mathrm {STT}}$ and large $j_e^{\mathrm {SOT}}$: in this case STT aids SOT acting as external field to bias the final magnetisation direction;
2. Intermediate $j_e^{\mathrm {STT}}$ and large $j_e^{\mathrm {SOT}}$: the system behaves as described in the previous point
3. Intermediate $j_e^{\mathrm {STT}}$ and small $j_e^{\mathrm {SOT}}$: in this case SOT alone cannot complete the reversal. Here SOT provides the initial tilt of the magnetisation to initiate the precessional dynamics during the 1 ns it is on. Once $j_e^{\mathrm {SOT}}$ is switched off, the magnetisation follows the spin polarised induced magnetisation dynamics.
4. Large $j_e^{\mathrm {STT}}$: here the reversal occurs in less than a nanosecond with SOT due to reduction of the incubation time through the SOT-induced initial torque assistance to the STT.

The following analysis of the magnitudes of the current densities shows that both conditions 1 and 4 are not efficient in terms of energy requirements. Those could be employed in high speed devices where the power consumption is not an issue. On the other hand, the scenario depicted in 3 would reduce the total switching time without increasing the energy requirements. Spin transfer torque switching assisted by spin orbit torque could prove useful for applications, in particular those where the balance between the efficiency and the power consumption is essential, such as in portable and smart devices.

We compute the write energy consumption $E_{\mathrm {w}}$, i.e. the energy required to reverse the magnetisation of the MTJ. $E_{\mathrm {w}}$ is obtained by summing the contributions from the MTJ and the HM:

1 $$\begin{aligned} E_{\mathrm {w}} = \int _{t_i}^{t_f} dt \left[ I_\mathrm {MTJ}(t)^2 R_\mathrm {MTJ}(t) + I_\mathrm {HM}(t)^2 R_\mathrm {HM}(t) \right] , \end{aligned}$$

where the integration is carried over the whole pulse duration $t_f - t_i$. $I_\mathrm {MTJ}(t)$ and $I_\mathrm {HM}$ are the electrical currents in the MTJ and in the HM at time *t*, respectively; similarly $R_\mathrm {MTJ}(t)$, $R_\mathrm {HM}(t)$ are the resistances of the MTJ and the HM. $R_\mathrm {MTJ}(t)$ is related to the gradient of the spin accumulation and spin current, and can be obtained from the resistance-area product $RA_\mathrm {MTJ}$^59,60^:

2 $$\begin{aligned} RA_\mathrm {MTJ} = \sum _i R_\mathrm {MTJ}A^i = \sum _i \frac{|\Delta m| a_0^2 t k_{\mathrm {B}} T }{j_m ^\mathrm {tot} e^2} \,. \end{aligned}$$

$RA_\mathrm {MTJ}^i$ is the resistance-area product at position *i*, $\Delta m$ is the difference of spin accumulation across the layers, $j_m ^\mathrm {tot}$ is the total spin current within the system, $a_0$ is the lattice constant of the material, *t* is the thickness of the regions the system is discretised into, *e* is the electron charge and $k_{\mathrm {B}} T = {10}\text { meV}$ is the electronic temperature. This temperature is the electronic temperature at which ab initio calculations of the density of states of minority and majority spin populations, used to determine the equilibrium spin accumulation $m_{\infty }$, are performed^60,61^. $j_m ^\mathrm {tot}$ accounts for both STT and SOT, hence during the initial 0.5 ns where both currents are applied $j_m ^\mathrm {tot}$ includes the contribution $j_m ^\mathrm {SOT}=\vartheta _{\mathrm {SH}} j_e^{\mathrm {SOT}}$.

**Figure 5:**
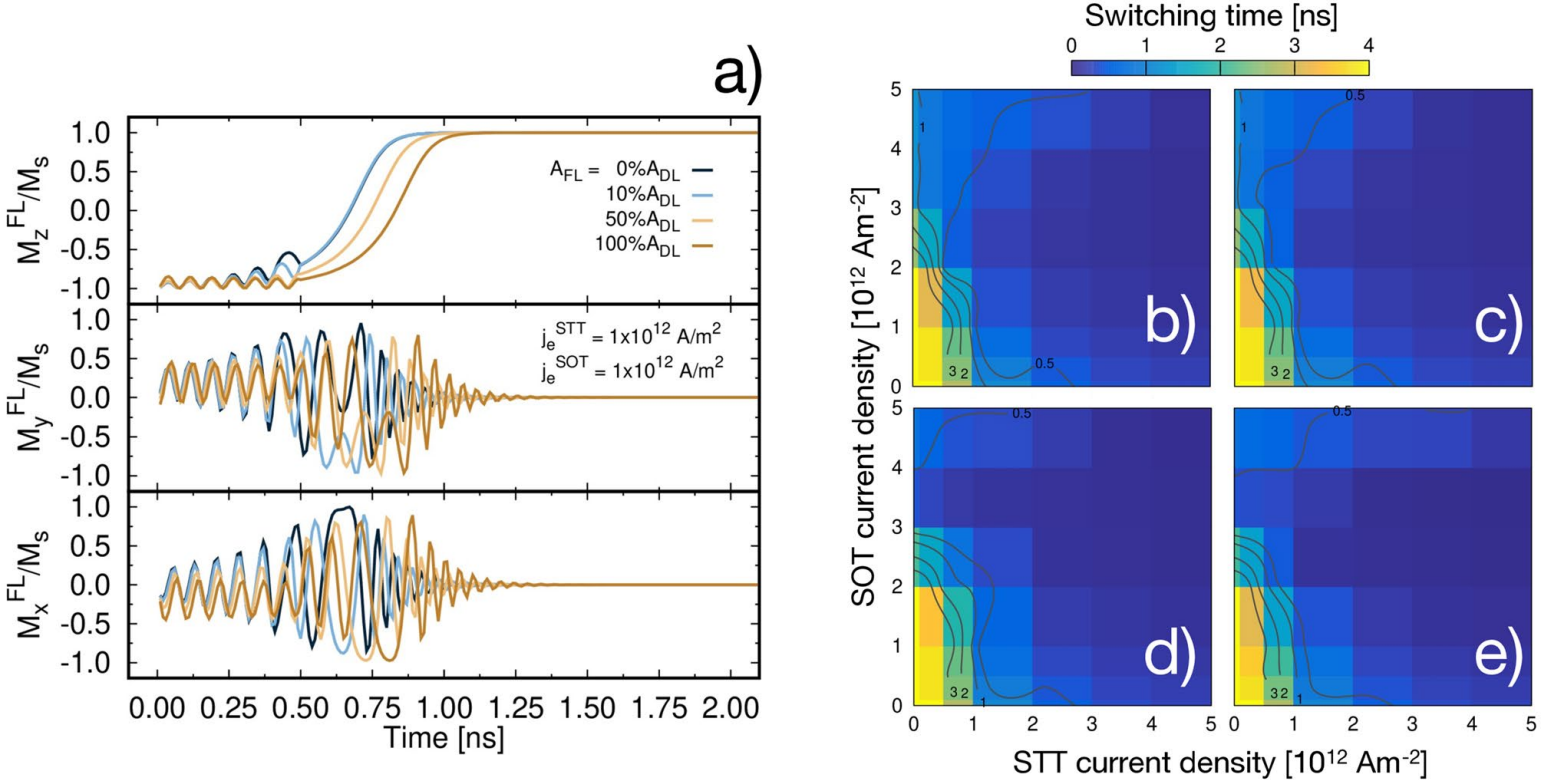
(**a**) Time evolution of the magnetisation components as a function of $A_{\mathrm {F}}$ for $j_e^{\mathrm {SOT}} =j_e^{\mathrm {STT}} ={1\times 10^{12}} \; \text {Am}^{-2}$ for a 20 nm MTJ. Plot of total switching time (colour) as a function of $j_e^{\mathrm {STT}}$ and $j_e^{\mathrm {SOT}}$ for $A_{\mathrm {F}}$ = 0, 0.1, 0.5 and 1 $A_{\mathrm {D}}$ for a 20 nm MTJ in panel (**b**–**e**) respectively. The colour scheme shows the switching time from immediate switching (blue) to no switching (yellow) within 4 ns. Dark grey lines represent mark points with switching time 0.5, 1, 2 and 3 ns.

The resistance of the HM contact $R_\mathrm {HM}(t)$ can be obtained from the resistivity $\rho _\mathrm {HM}$ of the HM as $R_\mathrm {HM} = \rho _\mathrm {HM} L_y / (L_x L_z)$, where $L_y$ is the length of the contact for $j_e^{\mathrm {SOT}}$ injected along *y* and $L_x L_z$ is the cross-sectional surface crossed by the electrons^35^. For Ta if we assume $\rho _\mathrm {HM} \sim {200} \; \upmu \Omega \text {cm}$^62^ and take a HM contact of dimensions ${50\times 50\times 4} \; \text { nm}$, we obtain $R_\mathrm {HM} \sim {500} \; {\Omega }$. In Fig. 4b we present the write energy for a 20 nm MTJ with $A_{\mathrm {F}} =A_{\mathrm {D}}$, corresponding to the dynamics shown in Fig. 4a. With the assistance of the SOT generated by the in-plane current $j_e^{\mathrm {SOT}}$, the magnetisation of the free layer can be reversed faster, but the average power dissipation of MTJ is greatly increased. However, when we consider the energy consumption of the MTJ related to the magnetisation reversal, i.e. neglecting other effects such as Joule heating, it can be clearly seen that low write energy can be achieved by optimising $j_e^{\mathrm {STT}}$ and $j_e^{\mathrm {SOT}}$. The obtained write energy is of the order of hundreds of fJ, in agreement with the results reported in literature for this kind of devices and also with technological requirements^9,10,34,35,42,45,46,63,64^. Increasing $j_e^{\mathrm {SOT}}$ results in high energy consumption, whereas the combination of SOT and STT currents assists in the reduction of write energy. Interestingly, for magnetisation switching solely driven by STT current, low energy consumption can be observed at high current density. This can be explained by considering that high current density significantly reduces the switching time yielding low writing energy. However, in practice it is difficult to generate high current density in a dot of few nanometre diameter without causing large Joule heating. The results suggest that the combination of low current density of SOT and STT can be used for a sub-nanosecond switching with low writing energy. This can be seen in panel c) of Fig. 4, where the product of write energy with switching time is plotted versus $j_e^{\mathrm {STT}}$ and $j_e^{\mathrm {SOT}}$. The diagram clearly shows how $j_e^{\mathrm {STT}} \sim {\sim 1.2\times 10^{12} \; \text {Am}^{-2}}$ and $j_e^{\mathrm {SOT}} \lesssim {1\times 10^{12}} \; \text {Am}^{-2}$ yield a switching that consumes 100fJ or less and it occurs in less than a nanosecond. More generally, such a diagram can prove useful to choose the most suitable switching schemes. Further, we consider the effect of different strengths of $A_{\mathrm {F}}$. To analyse this, we consider $j_e^{\mathrm {SOT}} =j_e^{\mathrm {STT}} ={1\times 10^{12}} \; \text {Am}^{-2}$, presented in Fig. 5a. As discussed for the case of pure SOT and taking into consideration Eq. (11), $A_{\mathrm {F}}$ acts on both precession and relaxation. Accordingly, we see a reduction in the switching time for weaker contributions of $A_{\mathrm {F}}$, with the fastest dynamics occurring for zero $A_{\mathrm {F}}$. The amplitude of the in-plane components of the magnetisation decreases as $A_{\mathrm {F}}$ increases and when SOT is turned off the reversal is delayed. Thus, weak SOT contribution to the precessional motion can yield better performance, with the limit case of zero $A_{\mathrm {F}}$. However, this condition does not represent the most physical case for a structure such as the MTJ investigated here, where interfaces are not equivalent and the stack is not symmetric. Instead, it is reasonable to expect a non-zero field-like contribution. In Fig. 5b–e we present the total switching time as a function of both $j_e^{\mathrm {STT}}$ and $j_e^{\mathrm {SOT}}$ for different $A_{\mathrm {F}}$ strength. From the phase plot we can see a large spectrum of combination of $j_e^{\mathrm {STT}}$ and $j_e^{\mathrm {SOT}}$ to yield the desired switching time. By comparing the contour plots for different $A_{\mathrm {F}}$ we can see how, as $A_{\mathrm {F}}$ increases, faster switching is achieved in the region 2 to ${4\times 10^{12}} \; \text {Am}^{-2}$. In particular we see that the fastest switching time achievable by applying both $j_e^{\mathrm {SOT}}$ and $j_e^{\mathrm {STT}}$ below ${1\times 10^{12}} \; \text {Am}^{-2}$ is 1 ns, independently of $A_{\mathrm {F}}$.

**Figure 6:**
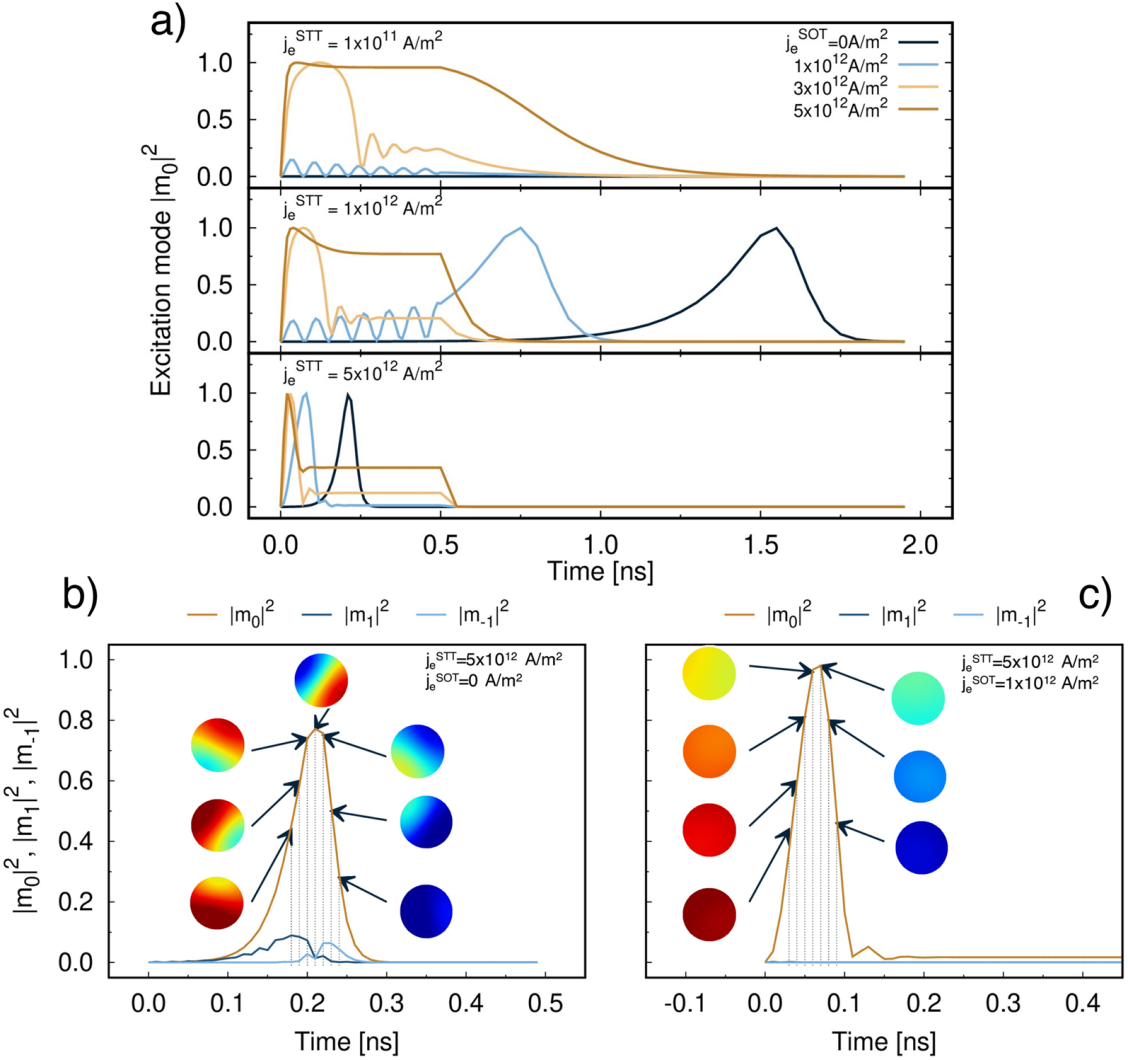
(**a**) Calculated amplitudes of the coherent excitation mode $m_0$ of the free layer of a 20 nm MTJ as a function of $j_e^{\mathrm {SOT}}$ for different $j_e^{\mathrm {STT}}$ and $A_{\mathrm {F}} =0.5A_{\mathrm {D}}$. (**b**) and (**c**) show the calculated amplitudes of $m_0$, $m_1$ (vortex) and $m_{-1}$ (antivortex) modes of a 30 nm MTJ FL for $A_{\mathrm {F}} =0.5A_{\mathrm {D}}$, $j_e^{\mathrm {STT}} ={1\times 10^{12}} \; \text {Am}^{-2}$ and $j_e^{\mathrm {SOT}} =0$, ${1\times 10^{12}} \; \text {Am}^{-2}$, respectively. Insets show snapshots of the *z*-component of the magnetisation with palette blue (+ 1), green (0), red (− 1).

Finally we investigate the reversal mechanism resulting from the combined application of STT and SOT to the system to evaluate the differences from the individual cases. The analysis involves calculation of the lowest-frequency modes ($w=0,1,-1$) defined in Eq. (12). When we combine the two effects, we observe different dynamics depending on the relative strengths of $j_e^{\mathrm {STT}}$ and $j_e^{\mathrm {SOT}}$. As expected, for diameters up to 20 nm the reversal is coherent with rotation of the in-plane components of the FL magnetisation. This is confirmed by the coherent mode $m_0$ being the only non-zero excitation mode. This is shown in Fig. 6a where the time evolution of the $m_0$ amplitude is plotted for $A_{\mathrm {F}} =0.5 A_{\mathrm {D}}$ as a function of different $j_e^{\mathrm {STT}}$ and $j_e^{\mathrm {SOT}}$. For large $j_e^{\mathrm {SOT}}$ $|m_0|^2$ reaches the maximum in about 100ps, subsequently it retains a finite value. The height of these plateaus increases with $j_e^{\mathrm {SOT}}$ followed by a decrease to zero after 0.5 ns, when $j_e^{\mathrm {SOT}}$ is turned off. The excitation modes we consider are deviations from the uniform state, i.e. magnetisation aligned along the easy axis ($\hat{z}$). Thus, a finite value is indicative of the incomplete switching, with the magnetisation uniformly aligned in a direction with an angle from the *z*-axis. Conversely, increasing the magnitude of $j_e^{\mathrm {STT}}$ for a fixed $j_e^{\mathrm {SOT}}$ corresponds to a decrease in the height of these constant regions. Thus, a stronger $j_e^{\mathrm {STT}}$ aids the reversal of the magnetisation, as also visible from Fig. 4a. The persistence of $m_0$ during the whole STT + SOT pulse shows how SOT is able to keep the system in an out-of-equilibrium excited state when $j_e^{\mathrm {SOT}}$ is large. Hence, to increase the energy efficiency, the pulse should be kept as short as possible for large $j_e^{\mathrm {SOT}}$. For a diameter of 30 nm, in the absence of $j_e^{\mathrm {SOT}}$ the reversal becomes non-collinear. However, the curves in Fig. 6a remain substantially unchanged for a diameter of 30 nm and hence this is not shown. Interestingly $j_e^{\mathrm {SOT}}$ tends to counteract the formation of non-collinear configurations. This effect is proportional to the magnitude of $j_e^{\mathrm {SOT}}$ and can be understood considering that SOT tends to favour in-plane magnetic configurations. Let us consider $j_e^{\mathrm {STT}} ={5\times 10^{12}} \; \text {Am}^{-2}$. For $j_e^{\mathrm {SOT}} ={0} \; \text {Am}^{-2}$ the reversal is non-collinear despite $m_0$ being the largest excited mode, as can be seen in Fig. 6b. As $j_e^{\mathrm {SOT}}$ increases $m_{1,-1}$ modes are suppressed and the reversal becomes more coherent. For $j_e^{\mathrm {SOT}} \ge {1\times 10^{12}} \; \text {Am}^{-2}$ $m_0$ is the only mode with non-zero amplitude, as shown in Fig. 6c. The change in reversal mechanism is also observed in the snapshots of the *z*-component of the FL magnetisation in these same figures. While for pure STT we can clearly observe the formation of a domain at the edge of the disk that propagates through the system accompanied by rotation around the *z*-axis, the magnetic texture becomes uniform for $j_e^{\mathrm {SOT}} ={1\times 10^{12}} \; \text {Am}^{-2}$. Similarly to what was already observed for the 20 nm diameter, further increase in $j_e^{\mathrm {SOT}}$ results in $m_0$ remaining excited until $j_e^{\mathrm {SOT}}$ is turned off. Different $A_{\mathrm {F}}$ strengths do not affect the reversal mechanism significantly and this is true for all diameters investigated. The main effect is the over-damped dynamics that quenches the precession of the in-plane components and causes a different switching time, as already observed above. Our results suggest that for small MTJ diameters even a weak $j_e^{\mathrm {SOT}}$ is able to make the reversal coherent. To induce non-uniformities such as nucleation processes in this combined STT-SOT system larger diameters^39,45,46^ and/or strong current densities would be required. As such, our assumption of spatially uniform current densities should not impact the results presented here.

In Ref.^35^ switching around 1 ns for $j_e^{\mathrm {SOT}} \sim 2.5\times 10^{11} \; \text {Am}^{-2}$ is achieved in STT-MTJs via SHE assist. The simulations are performed by means of a macrospin approximation and a relatively low anisotropy field $\sim {1}\text { T}$ is used, thus allowing for switching at lower current densities. Wang et al.^38^ experimentally obtain field-free switching in three terminal CoFeB/MgO-based perpendicular MTJs via interplay of STT and SOT. They also perform macrospin simulations including damping and field-like contributions for both STT and SOT. They find that $A_{\mathrm {F}}$ results in a reduction in $j_e^{\mathrm {SOT}}$ required to achieve reversal in contrast with our result where, depending on the relative magnitude between $j_e^{\mathrm {STT}}$ and $j_e^{\mathrm {SOT}}$, $A_{\mathrm {F}}$ contributions can either hinder or assist the reversal. We attribute the difference in the behaviour to the sign, and thus symmetry, of the damping-like torque ($-\varvec{m} \times \varvec{m} \times \varvec{\hat{\sigma }}$) and field-like torque ($\varvec{m} \times \varvec{\hat{\sigma }}$) within the macrospin model used by Wang et al. instead of the conventional formalism^11^. An analogous argument can be applied to the simulations performed in Ref.^40^ where a toggle MRAM is proposed. Our results exhibit similar features to those presented in Ref.^41^ by Pathak et al. However, we have investigated the effect of field-like contributions in SOT which were not considered in Ref.^41^ and we have found that a system where the field-like torque is minimised yields faster switching, in particular for larger $j_e^{\mathrm {SOT}}$. Moreover, the spin accumulation includes these contributions naturally for STT, and thus it allows for a more complete treatment of the phenomenon. In addition, Pathak et al. attribute the initial tilt of the magnetisation required to provide a non-zero torque to the presence of an Oersted field. While this can justify the initial tilt of the magnetisation in case of pure STT, we argue that the Oersted contribution should be negligible in case of SOT. In our approach we do not include such a contribution and in case of STT at 0K we initialise the FL magnetisation at an angle of ${1}^{\circ }$ from the z-axis to ensure a non-zero torque acts on the magnetisation. However, SOT symmetry ($\hat{\mathbf{S}}\times \varvec{\hat{\sigma }}$) is such that when the FL magnetisation has an out-of-plane component there is always a non-vanishing torque^38^, even for $A_{\mathrm {F}} =0$. This is evident from Eq. (8) and it also represents one of the main advantages of SOT. We can estimate the Oersted contribution due to $j_e^{\mathrm {SOT}} ={1\times 10^{12}} \; \text {Am}^{-2}$ from the Biot-Savart law to be $\lesssim {0.01}\text { T}$ for our geometry, although this varies both in magnitude and direction; for the same $j_e^{\mathrm {SOT}}$ the SOT fields are about an order of magnitude larger. Thus, importantly we expect the Oersted field contribution to be negligible in a combined STT–SOT dynamics since SOT provides the initial tilt of the magnetisation avoiding long incubation times. It is worth noting that at operational temperatures this contribution could be a source of Joule heating, as suggested in Ref.^65^. In Ref.^43^ Zhang et al. apply a combination of SOT and STT in a current-in-plane configuration with an in-plane MTJ, whereas we aim to exploit perpendicular MTJ since it is already working for pure STT dynamics. Sato et al.^39^ report a two-terminal SOT-MRAM cell based on a CoFeB/MgO MTJ. In this device the authors find that the switching mechanism is dominated by SOT and that STT offers little assist. Moreover, the setup requires an external in-plane magnetic field and the MTJ diameter is 110 nm. Such a MTJ dimension is characterised by non-coherent magnetisation dynamics, something undesirable in such applications, and is not feasible for high storage applications. Grimaldi et al.^45^ and Krizakova et al.^46^ demonstrate very promising SOT-induced switching assisted by STT and VCMA in three terminal CoFeB-based MTJs. However, to achieve a deterministic reversal with 100% switching probability an external in-plane magnetic field is applied in the case of SOT-induced dynamics. This is avoided in Ref.^46^ thanks to the stray field generated by an in-plane ferromagnetic layer embedded in the hard mask used for the SOT line, using a similar expedient to the one employed in Ref.^32^. In both studies however the relatively large dimension ($>{50}\;\text {nm}$) of the MTJ comports domain-wall mediated magnetisation dynamics, and this appear as incubation time even for pure SOT dynamics affecting the speed and energy consumption of the device. Moreover, if on the one hand having the interplay of SOT, STT and VCMA allows for more available configurations and solutions, on the other it makes the setup and control of these structures more complex than in the MTJ proposed in the our work.

We would like to stress that the simulations performed in the aforementioned works are obtained via either macrospin or micromagnetic approaches. Differently from these, the atomistic approach allows to account for the localised nature of the anisotropy, which arises at the interface in CoFe/MgO systems, as well as of the damping, as discussed in Section and shown in Table 1. In addition, since the thickness of the ferromagnetic layers in state of the art devices is of the order of a nanometre, even fine micromagnetic discretisation cells of ${1} \;\text {nm}^{3}$ are not able to resolve the dynamics of both magnetisation and, where considered, spin accumulation across the thickness accurately. In particular a macrospin approach assumes uniform magnetisation in the whole ferromagnetic layer, and as a consequence it cannot resolve non uniform magnetisation configurations such as those shown in Fig. 6, which can occur in device dimensions of technological relevance. Atomistic approaches can instead provide this level of detail and become the most appropriate choice to deal with such complex and miniaturised systems. Furthermore, we have investigated systematically the impact of combined STT and SOT on the reversal mechanism. SOT suppresses the non-uniform excitation modes at large dimensions and even for a small $j_e^{\mathrm {SOT}}$ the reversal becomes coherent. However this comes at the cost of maintaining the FL magnetisation in a non-equilibrium state until the SOT pulse is turned off.

## Discussion and conclusions

We have developed a model that combines atomistic spin dynamics self-consistently with spin transport models to investigate the miniaturisation necessary for novel MTJ design. By means of this approach we have modelled and investigated the magnetisation dynamics resulting from the combined action of spin orbit torque and spin transfer torque in CoFeB/MgO MTJs. By controlling $j_e^{\mathrm {STT}}$ and $j_e^{\mathrm {SOT}}$ the switching of the free layer magnetisation can be achieved in different regimes. Large $j_e^{\mathrm {SOT}}$ yields fast switching dynamics, where the SOT induced magnetisation dynamics is aided by the STT and field-free switching is achieved. However, in this case there is the downside of a high power consumption. For low $j_e^{\mathrm {SOT}}$ the SOT provides the initial tilt of the magnetisation assisting the STT dynamics with the possibility of reducing incubation time issues. Depending on the relative strength of $j_e^{\mathrm {SOT}}$ and $j_e^{\mathrm {STT}}$, the magnetisation follows a dynamical path which is closer to either that of STT or SOT or to a hybrid of both. Moreover, the reversal is characterised by coherent dynamics in presence of a non-zero $j_e^{\mathrm {SOT}}$ up to MTJ diameters of 30 nm, attributed to the symmetry of SOT that tends to suppress higher excitation modes. This limits the possibility of pinning of the magnetic texture and avoid stochasticity of the switching process. Our results highlight an intermediate regime with moderate $j_e^{\mathrm {STT}}$ and low $j_e^{\mathrm {SOT}}$ where the SOT assist the STT dynamics yielding switching below the nanosecond by reducing the transient time. Such a configuration of STT+SOT could result in improved device performances without affecting the energy consumption.

## Methods

### Atomistic spin model

In this work we use an atomistic spin model as implemented in the open source software package vampire^66,67^. The magnetisation dynamics of the system is modelled by integrating the stochastic Landau-Lifshitz-Gilbert (sLLG) equation of motion^68^:

3 $$\begin{aligned} \frac{d \hat{\mathbf{S}}_i}{dt} = - \frac{ \gamma }{\left( 1+\lambda ^2 \right) }\left[ \hat{\mathbf{S}}_i \times \mathbf {B^i_{{eff}}} + \lambda \hat{\mathbf{S}}_i \times \left( \hat{\mathbf{S}}_i \times \mathbf {B^i_{{eff}}} \right) \right] , \end{aligned}$$

where $\hat{\mathbf{S}}_i$ is the normalised unit spin vector on site *i*, $\gamma =1.76086\times 10^{11}\text {s}^{-1}\text {T}^{-1}$ is the gyromagnetic ratio of the electron and $\mathbf {B^i_{{eff}}}$ is the net effective field acting on the spin *i*. $\mathbf {B^i_{{eff}}}$ is obtained as:

4 $$\begin{aligned} \mathbf {B^i_{{eff}}} = - \frac{1}{\mu _{\varvec{s}} ^i} \frac{\partial \mathcal {H}}{\partial \hat{\mathbf{S}}_i} + \varvec{\xi _i} . \end{aligned}$$

The first term of Eq. (4) accounts for all the contributions in the localised extended Heisenberg Hamiltonian ($\mathcal {H}$):

5 $$\begin{aligned} \mathcal {H} = - \sum _{i < j} J_{ij} \hat{\mathbf{S}}_i\cdot \hat{\mathbf{S}}_j - \sum _{i} k_{\mathrm {u}} ^i ( \hat{\mathbf{S}}_i\cdot \hat{\varvec{e}})^2 - \sum _i \mu _{\varvec{s}} ^i \hat{\mathbf{S}}_i \cdot \mathbf {B_{{app}}} + \mathcal {H} _{\mathrm {dmg}} . \end{aligned}$$

where $J_{ij}$ is the exchange coupling constant between spins *i* and *j*, $k_{\mathrm {u}} ^i$ is the uniaxial energy constant on site *i* along the easy-axis $\hat{\varvec{e}}$, $\mu _{\varvec{s}} ^i$ is the atomic spin moment on the atomic site *i* and $\mathbf {B_{{app}}}$ is the external magnetic field. The magnetostatic contribution $\mathcal {H} _{\mathrm {dmg}}$ is calculated using a modified macrocell approach^69^ based on the work of Bowden et al.^70^. The second term of Eq. (4) couples the spin system with the heat bath via the the atomistic Gilbert damping parameter $\lambda$. For processes occurring on the nanosecond time-scale the thermal field $\varvec{\xi }$ can be described as a white noise term represented by a 3D Gaussian distribution whose width depends on temperature and $\lambda$^71^.

The spin transfer torque induced by the injection of spin polarised electrons into a ferromagnet is described following the spin-accumulation model including the contribution of adiabatic and non-adiabatic torques^60,61,72^. A spin polarised current builds a spin accumulation ($\mathbf {m}$), an imbalance between the population of spin-up and spin-down electrons, when it crosses a ferromagnetic material. The exchange interaction between the *s* spin polarised electrons and the *d* electrons of the ferromagnet results in a transfer of angular momentum. This interaction can be described as:

6 $$\begin{aligned} \mathcal {H} _{\mathrm {STT}} = -J_{\mathrm {sd}} \mathbf {m} \cdot \hat{\mathbf{M}}, \end{aligned}$$

where $J_{\mathrm {sd}}$ is the *s*–*d* exchange interaction, $\mathbf {m}$ is the spin accumulation and $\hat{\mathbf{M}}$ is the unit vector magnetisation of the ferromagnet. The evolution of $\mathbf {m}$ occurs on a faster time-scale, about 1ps, than the magnetisation, around 100ps, for the processes we are interested in. This allows to decouple the dynamics of the two: first solving for $\mathbf {m}$ assuming the magnetisation texture constant, and then calculating the torque via $\mathcal {H} _{\mathrm {STT}}$, which can be included in the Hamiltonian of Eq. (5) and as such it enters the LLG equation via $\mathbf {B^i_{{eff}}}$. The dynamics of the system drives $\mathbf {m}$ towards an equilibrium value $m_{\infty }$, defined as the difference between the equilibrium populations of spin-up and spin-down electrons^60^. $m_{\infty }$ is a characteristics of the materials and can be obtained from the density of states of majority and minority spin populations ($\propto N^{\uparrow } - N^{\downarrow }$)^60,73^, for instance by means of ab initio calculations. These calculations, as those reported in^60^, are often performed at an electronic temperature of 10 meV. The spin accumulation can be conveniently calculated in a rotated coordinate system where the unit vector components $\hat{\varvec{b}}_1$ and $\hat{\varvec{b}}_{2,3}$ are aligned parallel and perpendicular to the magnetisation direction respectively. The general solution of spin accumulation can be split into two parts: the longitudinal (${\varvec{m}_{\parallel }}$) and transverse (${\varvec{m}_{\bot }}$) components given by:

7 $$\begin{aligned} {\varvec{m}_{\parallel }}(x)= & {} \left[ {{m}_{\parallel }}(\infty ) + \left[ {{m}_{\parallel }}(0)-{{m}_{\parallel }}(\infty ) \right] e^{- x/{\lambda _\mathrm {sdl}}} \right] \hat{{\varvec{b}_1}} \nonumber \\ {\varvec{m}_{\bot , 2}}(x)= & {} 2e^{-k_1x}\left[ u \cos (k_2x)-v \sin (k_2x) \right] \hat{{\varvec{b}_2}} \nonumber \\ {\varvec{m}_{\bot ,3}}(x)= & {} 2e^{-k_1x} \left[ u \sin (k_2x)+v \cos (k_2x) \right] \hat{{\varvec{b}_3}}, \end{aligned}$$

where $(k_1 \pm ik_2) = \sqrt{\lambda _\mathrm {sf}^{-2} \pm i \lambda _{J}^{-2}}$, ${\lambda _\mathrm {sdl}}$ is the spin diffusion length, ${m}_{\parallel }(\infty )$ is the equilibrium value of spin accumulation, the spin-flip length is defined as ${\lambda _\mathrm {sf}} = \sqrt{2{D_0}{\tau _{sf}}}$ and ${\lambda _\mathrm {J}}$ is the spin-precession length. The unknown variables *u*, *v* and $m_{\Arrowvert }(0)$ can be evaluated from the boundary condition of the continuity of the spin current $j_m$.

The SOT contribution is introduced as an additional term to in LLG equation, following a similar approach to Slonczewski^74^ for STT. If we assume a bulk origin of the phenomenon, hence SOT induced by spin Hall effect (SHE), we can write:

8 $$\begin{aligned} \frac{d \hat{\mathbf{S}}_i}{dt} & = -\gamma \left( \hat{\mathbf{S}}_i \times \mathbf {B^i_{{eff}}} \right) + \lambda \left( \hat{\mathbf{S}}_i \times \frac{d \hat{\mathbf{S}}_i}{dt} \right) \\ & \quad - A_{\mathrm {D}} \hat{\mathbf{S}}_i\times \left( \hat{\mathbf{S}}_i \times \varvec{\hat{\sigma }} \right) - A_{\mathrm {F}} \left( \hat{\mathbf{S}}_i \times \varvec{\hat{\sigma }} \right) , \end{aligned}$$

where $\varvec{\hat{\sigma }}$ is the spin polarisation unit vector, $A_{\mathrm {D}}$ and $A_{\mathrm {F}}$ are the SHE spin orbit torque parameters that determine the strength of the damping- and field-like torque terms respectively. In writing Eq. (8), we have assumed that SOT can affect both the precessional dynamics and the relaxation process. It is fact not simple in structures such as an MTJ to discriminate and clearly separate these effects^11^. $\varvec{\hat{\sigma }}$ represents the direction of the polarisation of the spin current induced by the flow of electrons in the non magnet. $\varvec{\hat{\sigma }}$ is perpendicular to the electron flow, hence for a $j_e^{\mathrm {SOT}}$ flowing along the *y*-axis $\varvec{\hat{\sigma }}$ is directed both along *x* and *z*-direction. If the stack is piled along the *z*-direction, in first instance we can neglect the effect of $\varvec{\hat{\sigma }}$ along the same direction as there are not relevant interfaces. In this case $\varvec{\hat{\sigma }}$ can be expressed as:

9 $$\begin{aligned} \varvec{\hat{\sigma }} = \hat{\mathbf{z}} \times \hat{\mathbf{E}} , \end{aligned}$$

where $\hat{\mathbf{z}}$ is unit vector normal to the surface and $\hat{\mathbf{E}}$ is the electric field associated with $j_e^{\mathrm {SOT}}$. If we assume that the main origin of SOT is the spin Hall effect (SHE), the SOT parameters are given by:

10 $$\begin{aligned} A_{\mathrm {DL,FL}} = \frac{\hslash }{2e} \frac{V_{\mathrm {at}} j_e^{\mathrm {SOT}}}{\mu _{\varvec{s}} d} \vartheta _{\mathrm {SH}} = \frac{\hslash }{2e} \frac{j_e^{\mathrm {SOT}}}{M_{\mathrm {s}} d} \vartheta _{\mathrm {SH}}. \end{aligned}$$

Here, $\vartheta _{\mathrm {SH}}$ is the spin Hall angle which gives the conversion efficiency of electrical current into spin current, $d$ is the ferromagnet thickness, $V_{\mathrm {at}}$ is the atomic volume and $M_{\mathrm {s}} =\mu _{\varvec{s}}/V_{\mathrm {at}}$ is the magnetisation of the ferromagnet. This is not true generally, in particular for structures with structural inversion asymmetry^11^, where the Rashba or inverse spin galvanic effect (iSGE) should be accounted for. However, due to the complexity in determining the strength of the Rashba effect, we have decided to apply the aforementioned approximation. In presence of such SOT, the solved LLG equation can be expressed explicitly as:

11 $$\begin{aligned} \frac{d \hat{\mathbf{S}}_i}{dt} =&- \frac{\gamma }{\left( 1+\lambda ^2 \right) } \hat{\mathbf{S}}_i \times \mathbf {B^i_{{eff}}}- \frac{\gamma \lambda }{\left( 1+\lambda ^2 \right) } \left[ \hat{\mathbf{S}}_i \times \left( \hat{\mathbf{S}}_i \times \mathbf {B^i_{{eff}}} \right) \right] \nonumber \\&- \frac{\gamma \left( A_{\mathrm {F}}- \lambda A_{\mathrm {D}} \right) }{\left( 1+\lambda ^2 \right) } \left( \hat{\mathbf{S}}_i \times \varvec{\hat{\sigma }} \right) \nonumber \\&- \frac{\gamma \left( A_{\mathrm {D}} + \lambda A_{\mathrm {F}} \right) }{\left( 1+\lambda ^2 \right) }\left[ \hat{\mathbf{S}}_i \times \left( \hat{\mathbf{S}}_i \times \varvec{\hat{\sigma }} \right) \right] . \end{aligned}$$

Even though this form of the LLG equation is not computationally efficient, it demonstrates that if both damping-like and field-like contributions are included they intermix. If we consider an effective field $\mathbf {\tilde{B}^i_{{eff}}} = \mathbf {B^i_{{eff}}} + \mathbf {B^i_{{SOT}}}$, where $\mathbf {B^i_{{SOT}}} =A_{\mathrm {D}} \left( \hat{\mathbf{S}}_i \times \varvec{\hat{\sigma }} \right) + A_{\mathrm {F}} \varvec{\hat{\sigma }}$, the LLG equation with SOT can be written as Eq. (3) where $\mathbf {B^i_{{eff}}}$ is replaced by $\mathbf {\tilde{B}^i_{{eff}}}$.

### Investigated system

We model CoFeB as cylindrical alloy with a body-centred cubic (bcc) crystal structure with lattice constant 2.86Å. CoFeB/MgO is characterised by a large perpendicular single-ion uniaxial interfacial anisotropy with zero anisotropy in the bulk^75^. The CoFeB atomic layers opposite to MgO are assumed to have no particular interfacial properties and we neglect the small bulk anisotropy of CoFeB^7^. We use a mean field approximation that depends on the Curie temperature $T_{\mathrm {c}}$ to extract the atomistic exchange parameters $J_{ij}$. To extract interfacial and bulk $J_{ij}$ values we impose a unique $T_{\mathrm {c}}$ in the whole CoFeB, where $T_{\mathrm {c}}$ is taken from CoFeB/MgO thin film measurements^75^. We describe CoFeB as having an atomic spin moment $\mu _{\varvec{s}}$ of 1.6 $\mu _{\mathrm {B}}$, in agreement with experimental reports on similar systems^7^. The CoFeB/MgO interface has a larger damping than the rest of the CoFeB, in agreement with both experimental^7,76^ and theoretical^54^ works. Boron is not directly included in the simulations because it is not magnetic and because tends to diffuse out of the CoFe layer during annealing. The FL is capped by a heavy metal (HM) layer, which we take to be Ta. The role of HM is to convert electrical current into spin current that flows in the adjacent ferromagnet, whilst no magnetisation dynamics is assumed occurring within the material itself. Hence, we account for the effect of HM via the spin Hall angle $\vartheta _{\mathrm {SH}}$, that we assume 0.11 for Ta^34,77–79^, and we do not include it in the simulations explicitly.

### Normal modes analysis

In order to characterise the reversal process we employ the method of Visscher et al.^48,50^ based on the stability of the lowest frequency normal modes, which are a small perturbation of the equilibrium magnetic state. They classify these modes by the winding number *w*, an integer index that can be understood as the number of times the magnetisation of the disk winds around a symmetry axis perpendicular to the plane of the disk when the disk is revolved once. The amplitudes of the three lowest-frequency modes ($w=0,1,-1$) can be expressed as^48,50^:

12 $$\begin{aligned} m_{0}&= \int _{w} \left( s_x + i s_y\right) dxdy \nonumber \\ m_{1}&= \int _{w} \left[ \left( x s_x + y s_y \right) +i \left( x s_y - y s_x\right) \right] dxdy \nonumber \\ m_{-1}&= \int _{w} \left[ \left( x s_x - y s_y \right) +i \left( x s_y + y s_x\right) \right] dxdy \, , \end{aligned}$$

where *x*, *y*, $s_x$ and $s_y$ are the spatial and spin coordinates of the atoms within the system, respectively, *i* is the imaginary unit, *w* is the surface of the disk and *dxdy* is the surface element over which the integration is carried out. For the $w=0$ mode the magnetisation in nearly independent of position, hence we refer to this as the uniform mode. This is the lowest energy mode and is defined as the “coherent mode”. The other two modes represent the first excited states and are defined as “vortex” ($w=1$) and “antivortex” ($w=-1$).

## Data Availability

The datasets generated during and/or analysed during the current study are available from the corresponding author on reasonable request.
